# The Relationship Between Sensation Seeking and Tobacco and Alcohol Use Among Junior High School Students: The Regulatory Effect of Parental Psychological Control

**DOI:** 10.3389/fpsyg.2019.02022

**Published:** 2019-09-04

**Authors:** Weiguo Zhao, Fei Xu, Wen Ding, Yining Song, Qi Zhao

**Affiliations:** School of Psychology, Shandong Normal University, Jinan, China

**Keywords:** sensation seeking, parental control, tobacco and alcohol use, junior high school students, moderating effect

## Abstract

The present study primarily aims to examine differences in the use of tobacco and alcohol by junior high school students under different parental control levels (including parental psychological control and parental behavioral control). It thus explores the regulatory effect of parental control on the relationship between adolescent sensation seeking and tobacco and alcohol use. A total of 1,050 junior high school students in Shandong province were surveyed using sensation-seeking scale, parental control scale, and adolescent health-related risk behavior questionnaire. As the results showed, (1) sensation seeking and gender had effects on the use of tobacco and alcohol among junior high school students; (2) parental psychological control can enhance and moderate the relationship between sensation seeking and the use of tobacco and alcohol; (3) parental behavioral control cannot regulate the relationship between sensation seeking and the use of tobacco and alcohol among junior high school students; and (4) no significant urban-rural differences were found regarding the regulatory effects of parental psychological control on sensation seeking and alcohol and tobacco use in junior high school students.

## Introduction

In recent years, smoking and drinking among junior high school students (mostly aged 12–15) have become a public health issue attracting great concern. According to the China Youth Tobacco Survey Report, the smoking rate of junior high school students aged 13–15 was 6.4%:10.6% for boys and 1.8% for girls, respectively (China Centre for Disease Control, 2014). According to the 2018 Alcohol and Health Status Report published by the World Health Organization, more than a quarter (26.5%) of adolescents aged 15–19 are current drinkers (World Health Organization, 2018). This rebellious phenomenon is prominent due to the imbalance between the physiological maturity and psychological maturity of junior high school students (Han et al., 2016), which is likely to cause adolescents to violate their parents’ wishes and to engage in negative behaviors, such as smoking and drinking. Tobacco and alcohol use is very harmful for junior high school students, as it not only will damage their physical health by affecting the central nervous system (Benowitz et al., 2016), but also could trigger some behavioral and social problems such as interest addiction, depression, anxiety, and other adverse consequences (Visser et al., 2012; Huang et al., 2014). To prevent and control the tobacco and alcohol use in junior high school students, it is necessary to explore its influencing factors and its mechanism of action.

Previous studies have studied the effects of demographic variables such as grade, gender, and urban-rural residence on the externalized behavior of tobacco and alcohol use. First, previous studies have shown that there are significant gender differences in the use of tobacco and alcohol, and boys engage in more smoking and drinking behavior than girls (Xia et al., 2012; Yuan et al., 2016). Only a few studies have found that there are no significant gender differences in adolescent externalizing behavior (Wang et al., 2013). Therefore, we propose research Hypothesis 1a: there is a significant gender difference in the use of tobacco and alcohol in junior high school students, boys engage in more smoking and drinking behavior than girls. Second, studies have shown that there are urban-rural differences in the use of tobacco and alcohol and that the proportion of smoking and drinking behavior among rural middle school students is significantly higher than that among urban middle school students (Atav and Spencer, 2002; Zhi and Chen, 2008). Therefore, we propose Hypothesis 1b: there are significant urban-rural differences in the use of tobacco and alcohol in junior high school students, the smoking and drinking behaviors of rural middle school students were significantly higher than those of urban middle school students. Third, some studies have found that the differences in drinking behavior among young people between different grades are not significant (Lin et al., 2010). However, there are also studies that recruit students in the first, second, and third grades of junior high school as subjects that find significant grade differences in adolescents’ externalized problem behaviors. Specifically, it has been found that there are significantly more problems with externalized behavior in junior three than in junior one or junior two but that there is no significant difference in problems with externalized behavior between the first and second grades (Wang et al., 2013). This difference may be because junior high school students are facing more pressure from the senior high school entrance examination, which leads them to engage in more tobacco and alcohol use behavior to relieve stress. This study focuses on the influence of parental control on the use of tobacco and alcohol in junior high school students, and the pressure of the senior high school entrance examination is a variable that needs to be controlled. Therefore, third grade junior high school students are excluded, and Hypothesis 1c is proposed: there is no significant grade difference in the use of tobacco and alcohol by junior high school students. Finally, to lay the foundation for a comprehensive analysis and understand the influencing factors of tobacco and alcohol use, this study will first explore the relationship between demographic variables and tobacco and alcohol use and then further explore any contradictions brought to light.

Previous research has found that sensation seeking increased the risk of initiating substance use (Jensen et al., 2017). Many studies have shown that sensation seeking is a risk factor for adolescent tobacco and alcohol use (Wang et al., 2012b; Meil et al., 2016; Yuan et al., 2016). Sensation seeking refers to individuals engaging in social, physiological, economic, or legal risk-taking behaviors because they seek novel, intense, complex, and varied emotional experiences (Chen et al., 2006; Zuckerman, 2008). Nicotine in tobacco could stimulate the central nervous system (Benowitz et al., 2016), increases the amount of dopamine in the brain (Shiffman and Kirchner, 2009). Alcohol poisoning can cause irritability, hallucinations, and other adverse symptoms. Overall speaking，tobacco and alcohol use together can make individuals happier, thus promoting more smoking and drinking behaviors in individuals with high sensation seeking (Piasecki et al., 2011; Yuan et al., 2016). In addition, junior high school students have lower risk assessment ability, and thus it is easier for junior high school students who have higher sensation-seeking levels to underestimate the risk of tobacco and alcohol use, resulting in more tobacco and alcohol use behaviors (Hoyle et al., 2002; Romer and Hennessy, 2007). Therefore, sensation seeking is a risk factor for the use of tobacco and alcohol in junior high school students. We propose Hypothesis 2: sensation seeking significant positively predicted the use of tobacco and alcohol by junior high school students.

Although studies have investigated the impact of sensation seeking on problem behaviors such as tobacco and alcohol use by junior high school students, there has been less discussion about the mechanism of action between them. Social cognition theory emphasizes how individual cognition, behavior, and environmental factors and their interactions influence individual behavior (Bandura, 1997). Even individuals with the same perceptions who are living in different environments may exhibit different problems. In the family environment, parental control has an important influence on the use of tobacco and alcohol in junior high school students. Parental control refers to a relatively stable behavior used by parents to manage and control their children. Barber et al. (1994) first proposed the division of parental control into psychological control and behavioral control based on the parent’s control point source; that is, the classification criteria are based on the parent’s control over the child’s psychological or external behavior. Psychological control refers to parents trying to control their child’s behavior by controlling their emotions, thoughts, and parent-child relationship and disrupting their children’s autonomy (Wang et al., 2007; Arim and Shapka, 2008). Behavioral control refers to parents controlling the external behavior of adolescents by supervising, restricting, and establishing rules. The parent effect model argues that specific parenting behaviors, such as psychological control, can significantly influence the development of problem behavior among adolescents (Branje et al., 2008). Empirical studies have shown that there is a significant correlation between parental control and adolescent tobacco and alcohol use behavior (Ye et al., 2012; Lai et al., 2014a). However, the results of previous research on the effects of parental control of behaviors such as the use of tobacco and alcohol among adolescents are not consistent. Studies have found that psychological control has a negative impact on individual development and that parents’ psychological control can significantly predict the child’s externalization behavior (Arim and Shapka, 2008; Lansford et al., 2014; Xing et al., 2017; Chen et al., 2018). Wang et al. (2007) noted that parental behavioral control can promote appropriate development outcomes (such as reduced disciplinary behavior) among adolescents. Social connection theory can explain this conclusion, which holds that the parental supervision of adolescents is an important component of social connections. When adolescents are supervised by parents, the opportunities for them to be exposed to crimes or problematic behaviors will be reduced, which can also reduce the likelihood of these problem behaviors (Hirschi, 1969). In summary, parents’ behavioral control and psychological control have different effects on the behavior of junior high school students. Parental behavioral control can reduce the use of tobacco and alcohol among junior high school students, while parental psychological control can increase the use of tobacco and alcohol for junior high school students.

Studies have shown that parental regulation plays a moderating role in the relationship between socializing with problematic companions and adolescents’ own problem behaviors (Wang et al., 2014). Adolescent parental monitoring also plays a moderating role in the relationship between Internet addiction and social adaptation (Wang et al., 2011); thus, it can be seen that parental supervision and control may play a moderating role in many variables involved in adolescents’ adaptive behavior, but no research has examined the specific mechanism of parental control in the relationship between adolescents’ sensation seeking and the use of tobacco and alcohol. Junior high school students are experiencing puberty and in a semi-mature but also semi-naive mental state. The higher the level of parental psychological control is, the more easily it may provoke their rebellious psychology. These students may be more likely to violate their parents’ wishes and engage in smoking and drinking behaviors. Many studies have shown that parental psychological control can positively predict behavior such as the internalization of problems such as depression in adolescents (Lai et al., 2014b; Peng et al., 2016). Junior high school students with psychological depression are more likely to engage in smoking and drinking to relieve their inner grief (Xu and Huang, 2013). High levels of parental behavioral control may reduce the chances that junior high school students are exposed to tobacco and alcohol, thereby reducing their use of these substances. Therefore, we propose Hypothesis 3: parental control can moderate the relationship between sensation seeking and the use of tobacco and alcohol in junior high school students; Hypothesis 3a: parental psychological control can enhance the relationship between sensation seeking and tobacco and alcohol use in junior high school students; and Hypothesis 3b: parental behavioral control can weaken the relationship between sensation seeking and the use of tobacco and alcohol in junior high school students.

Most previous studies have directly explored the relationship between sensation seeking and internalizing problems (e.g., depression and anxiety) or externalizing problems (e.g., alcohol and tobacco use) (Hittner and Swickert, 2006; Ko et al., 2007; Gu et al., 2012). Some studies also used family, school, and other environmental variables as exterior factors when conducting research (Stephenson and Helme, 2006; Ye et al., 2011; Yuan et al., 2016; Zhao et al., 2017). Due to the immature psychological development of junior high school students, the phenomenon of rebellion is particularly prominent. It is particularly important to explore the role of parental control between sensation seeking and the tobacco and alcohol use, which has not been explored. Moreover, the differences between urban and rural areas in this model have not been explored. Before China began constructing the new countryside, its urban and rural areas were quite different. However, with the introduction of socialist modernization and the development of urban and rural integration, China’s rural areas have entered a period of transition. The spiritual, cultural, and cognitive levels of rural residents have developed significantly, and rural education levels have made great progress. To test the moderating effect of parental control, the path model map is suitable for different regions, that is, it can be used to test consistency in the effects of demographic variables on junior high school students. By examining the consistency of rural and urban issues in family education and junior high school students’ behavior in recent years and exploring the cultural and educational development gap between rural and urban areas, we further compare the moderation models. Sensation seeking is associated with novel and risky behaviors. Urban students have more adverse behaviors such as drug use than rural students, and teenagers living in urban environments have significantly higher levels of excitement seeking than those living in rural areas (Gordon and Caltabiano, 1996; Scherer et al., 2000). Therefore, to verify the existence of significant urban-rural differences in junior high school students’ use of tobacco and alcohol and to establish a moderating effect model of parental control on junior high school students’ sensation seeking and use of tobacco and alcohol, we will further propose Hypothesis 4: in the entire moderating model, there are significant differences between urban and rural areas, this moderating model maybe more applicable in cities than in rural areas.

In summary, we propose a moderation model based on the parental effect model and social connection theory. The following content reflects the main discussion: (1) investigate the relationship between sensation seeking and the use of tobacco and alcohol in junior high school students; (2) explore the moderating effect of parental control on sensation seeking and junior high school students’ use of tobacco and alcohol; (3) identify whether there are significant differences between urban and rural areas in the regulation model.

## Materials and Methods

### Participants

A total of 1,050 questionnaires were distributed in two ordinary junior high schools in Shandong province by cluster sampling. A total of 1,009 valid questionnaires were collected, for an effective recovery rate of 96.10%. The age range of the subjects was from 11 to 16 years, *M* = 13.34 years, SD = 0.71. There were 536 male students, accounting for 53.12% of the total, and 473 female students, accounting for 46.88% of the total number. The subjects covered the seventh and eighth grades of junior high school. There were 588 students in the seventh grade, accounting for 58.28% of the total, and 421 students in eighth grade, accounting for 41.72% of the total number. Examining family residence, 321 students lived in an urban area, accounting for 31.81% of the total, and 688 lived students in a rural area, accounting for 68.19% of the total.

### Measures

#### Sensation Seeking

Sensation seeking was measured using the Primary and Secondary Sensation-Seeking Scale (Chen et al., 2006), which includes the Disinhibition (Dis) subscale and the Excitement and Adventure Seeking (TAS) subscale. There are 15 items in each subscale, and the questionnaire uses a 3-point scale (1 = “do not want to do”; 2 = “want to do, but will not necessarily do”; 3 = “want to do if there is a chance to do it”). The scores of all items are added to obtain the original total score, and a higher total score for the subjects indicates a higher level of perceived sensation seeking. In this study, the Cronbach’s alpha coefficient was 0.898. The CFA test found that structural validity was good, *χ*^2^/df = 3.27, CFI = 0.91, TLI = 0.90, RMSEA = 0.05.

#### Parental Control

The Parental Control Scale (Wang et al., 2007) was used to measure the level of parental control. The scale consists of two subscales: the psychological control subscale and the behavioral control subscale. The psychological control subscale contains 18 items, such as “My parents say that if I truly care about them, I will not do things that make them worry.” The behavioral control subscale contains 16 items, such as “My parents took the initiative to talk to me about what I did with my friends.” The questionnaire uses five points to score, ranging from 1 = “very disagreeable” to 5 = “very consistent,” respectively. The higher the level of behavioral control is, the higher the score. In this study, the Cronbach’s alpha coefficients of the Parental Psychological Control and Behavior Control subscales were 0.96 and 0.92, respectively. The CFA test found that the structural validity was good, *χ*^2^/df = 3.41, CFI = 0.92, TLI = 0.90, RMSEA = 0.05.

#### Tobacco and Alcohol Use

The subscale for smoking and drinking behavior in the Adolescent Health Related Risk Behavior Questionnaire (Wang et al., 2012a) was used. The questionnaire consisted of six items that examined smoking and drinking behavior among adolescents. Each behavior consists of three items, such as “Do you smoke?” and “Have you ever had a drink or been drunk?”. The questionnaire is scored from 1 to 5 points ranging from “never” to “always.” The scores of smoking and drinking behaviors were added together to form the total scores of tobacco and alcohol use questionnaire. To reduce false answers from participants motivated by a desire for social approval, the formal test was conducted using a double anonymous approach (anonymity of the participants, anonymous questionnaire), and six unrelated items were added, such as “Do you have breakfast?,” which were not counted in the final score. In the present study, the Cronbach’s alpha coefficient of the questionnaire was 0.890. The CFA test found that the structural validity was good, *χ*^2^/df = 2.75, CFI = 0.99, TLI = 0.97, RMSEA = 0.04.

### Procedure

The survey was conducted during September 2018. The data were collected on the class as a unit, in junior high schools from Shandong province of China, by graduate students majoring in psychology who had received professional and systematic training. The process is about 40 min long, in a self-study class. In the process, we emphasized that survey data were kept confidential and were used for academic purposes only. The questionnaires completed by respondents were collected uniformly. This study has obtained the written informed consent of the students’ parents, as well as with the approval of the Institutional Review Board of Shandong Normal University and the target junior high schools.

### Data Analysis

The completed questionnaires were numbered and analyzed using SPSS Statistics 16.0 and AMOS 17.0 software. Pearson product-moment correlation analysis among three variables: sensation seeking, tobacco and alcohol use, and parental control was conducted by using SPSS16.0. Hierarchical regression analysis was used to examine the moderating effect of parental control in three steps: gender was included in the first step; sensation seeking and parental control were included in the second step; and binary interaction terms of sensation seeking and parental control were included in the third step. Then simple slope analysis was used to examine the direction of the moderating role of parental control. We use independent sample *t* test to examine the differences of the three main variables. Specifically, gender, urban and rural area, and grade variables were put into grouping variables respectively; sensation seeking, psychological control, behavior control, and tobacco and alcohol use were put into test variables respectively; and then the differences of the three variables in demographic variables were tested. In order to explore the applicability of the model in urban and rural areas, this study used AMOS 17.0 to conduct multiple comparisons. In the model comparison, we use the unconstrained model M1 and the constrained model M2. The unconstrained model is freely estimated for all parameters, and the constrained model is equalized by all regression coefficients.

## Results

### Common Method Deviation

Because all the variables in this study are from the self-reports of adolescents, there may be a common method deviation problem (Zhou and Long, 2004). Therefore, common method deviation was statistically confirmed by the Harman single factor test method. The results show that the first principal factor explained a variation of 13.298%, so the threat of common method deviation in this study is low, and the data can be analyzed in the next step.

### Descriptive Statistics

Taking the average score of the questionnaire items as an indicator, the average, standard deviation and correlation coefficient of each variable were obtained (see Tables 1, 2). The *t* test was used to investigate the gender differences for each variable. It was found that the psychological control of junior high school students and the use of tobacco and alcohol had significant gender differences (*t* = 2.355, *p* < 0.05; *t* = 3.416, *p* < 0.001), boys engage in more smoking and drinking behavior than girls, which supported Hypothesis 1a. There is no gender difference in sensation seeking and behavioral control among junior high school students. The *t* test was used to investigate urban-rural differences in the variables. There was a significant urban-rural difference between sensation seeking and tobacco and alcohol use (*t* = 5.529, *p* < 0.001; *t* = 3.299, *p* < 0.001), the smoking and drinking behaviors of urban middle school students were significantly higher than those of rural middle school students; this is not consistent with Hypothesis 1b, but there was no urban-rural difference between psychological control and behavioral control. The *t* test was used to investigate the grade differences for each variable. There were significant grade differences in sensation seeking, psychological control, and behavioral control (*t* = −3.277, *p* < 0.001; *t* = 3.046, *p* < 0.01; *t* = 2.505, *p* < 0.05). There was no grade difference in the use of tobacco and alcohol among junior high school students, which supported Hypothesis 1c.

**Table 1:** Means and standard deviations for the variables (*N* = 1,009).

Variables	Mean (SD)	1	2	3	4
Gender	Male Female	1.61 (0.34)	2.62 (0.72)	3.27 (0.77)	1.25 (0.45)
		1.64 (0.34)	2.50 (0.77)	3.23 (0.79)	1.17 (0.33)
Grade	Seven Eight	1.60 (0.35)	2.62 (0.72)	3.30 (0.78)	1.21 (0.39)
		1.67 (0.34)	2.48 (0.78)	3.18 (0.77)	1.22 (0.42)
Urban/rural	Urban Rural	1.71 (0.35)	2.54 (0.79)	3.26 (0.84)	1.27 (0.45)
		1.59 (0.33)	2.57 (0.73)	3.24 (0.75)	1.19 (0.38)

**Table 2:** Correlations for the variables.

	1	2	3	4
1. Sensation seeking	1			
2. Psychological control	0.12^**^	1		
3. Behavior control	0.11^**^	0.30^**^	1	
4. Tobacco and alcohol use	0.30^**^	0.18^**^	0.00	1

** *p < 0.01*.

Pearson correlation analysis of each study variable showed that sensation seeking was significantly positively correlated with psychological control, behavioral control, and the use of tobacco and alcohol. Psychological control as a form of parental control was significantly positively correlated with behavioral control, tobacco and alcohol use. Behavioral control was not significantly related to the use of tobacco and alcohol.

### The Predictive Effect of Sensation Seeking on Alcohol and Tobacco Use

This study provided statistical control of sensation seeking by incorporating the antecedent variable “sensation seeking” into the regression equation to obtain the predictive effect of sensation seeking on alcohol and tobacco use. The results showed that sensation seeking had a significant positive predictive effect on alcohol and tobacco use in junior high school students (*β* = 0.307, *t* = 10.232, *p* < 0.001) and could account for 9.4% of the variation. Therefore, Hypothesis 2 was supported: sensation seeking could positively predict the use of alcohol and tobacco in junior high school students.

### Analysis of the Moderation Role of Parental Control

Taking the use of tobacco and alcohol as the dependent variable, multiple regression analysis was used to investigate the relationship between sensation seeking and tobacco and alcohol use and the moderating effect of parental control. Because the two dimensions of parental control (psychological control and behavioral control) are reversed in the hypothesis, the multiple regression analyses are performed separately.

First, we explored the role of psychological control regulation. Each variable enters the regression model in three steps: the first step is to input gender as a control variable in the regression equation; the second step is to input sensation seeking and psychological control; and the third step is to input the binary interaction term (sensation seeking × psychological control). The results (see Table 3) showed that after controlling for the effect of gender, the interaction term of sensation seeking and psychological control could significantly predict tobacco and alcohol use (*β* = 0.464, *t* = 2.712, *p* < 0.01). Therefore, Hypothesis 3a was supported: psychological control can play a moderating role in the relationship between sensation seeking and the use of tobacco and alcohol.

**Table 3:** The relationship between sensation seeking and the use of tobacco and alcohol: the regulation of psychological control.

	*b*	SE	*β*	*t*	Δ*R*^2^
First step					0.011
Gender	−0.085	0.025	−0.105	−3.354^***^	
Second step					0.127
Gender	−0.084	0.024	−0.104	−3.543^***^	
Sensation seeking	0.339	0.035	0.288	9.735^***^	
Psychological control	0.094	0.016	0.174	5.882^***^	
Third step					0.006
Gender	−0.084	0.024	−0.103	−3.527^***^	
Sensation seeking	0.041	0.115	0.035	0.357	
Psychological control	−0.094	0.071	−0.174	−1.319	
Sensation seeking × psychological control	0.114	0.042	0.464	2.712^**^	

** *p < 0.01*.

*** *p < 0.001*.

Second, we explored the role of behavioral control regulation. Again, each variable enters the regression model in three steps: first, gender is entered into the model, followed by sensation seeking, and then, third, their binary interaction (sensation seeking × behavioral control). The results (see Table 4) showed that after controlling for gender, the interaction between sensation seeking and behavioral control did not predict tobacco and alcohol use (*β* = −0.037, *t* = −0.184, *p* > 0.05). Therefore, parental behavior control did not play a moderating role in the influence of sensation seeking on alcohol and tobacco use.

**Table 4:** The relationship between sensation seeking and the use of tobacco and alcohol: the regulation of behavioral control.

	*b*	SE	*β*	*t*	Δ*R*^2^
First step					0.011
Gender	−0.085	0.025	−0.105	−3.354^***^	
Second step					0.098
Gender	−0.096	0.024	−0.119	−3.989^***^	
Sensation seeking	0.370	0.035	0.314	10.505^***^	
Behavioral control	−0.015	0.015	−0.028	−0.945	
Third step					0.000
Gender	−0.097	0.024	−0.119	−3.988^***^	
Sensation seeking	0.396	0.148	0.337	2.671^**^	
Behavioral control	−0.001	0.076	−0.002	−0.013	
Sensation seeking × behavioral control	−0.008	0.045	−0.037	−0.184	

** *p < 0.01*.

*** *p < 0.001*.

**Figure 1:**
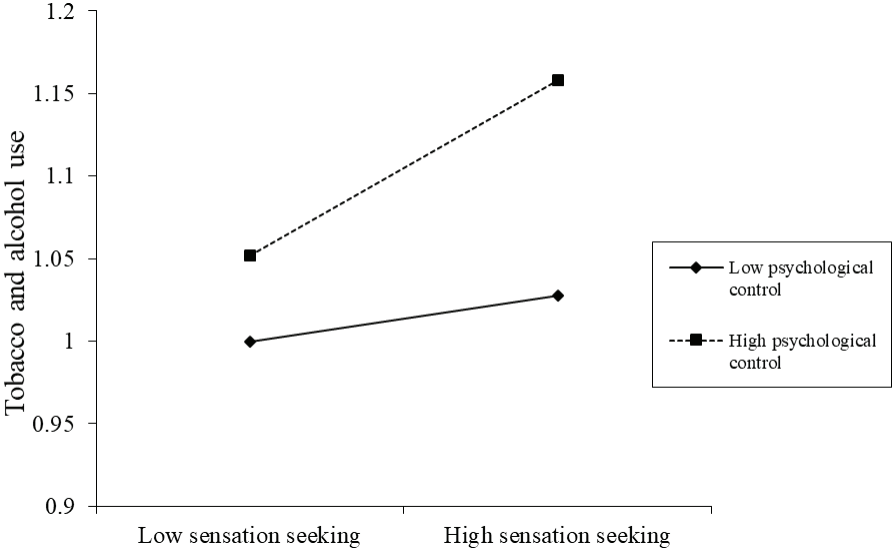
The moderating effect of psychological control on the relationship between sensation seeking and the tobacco and alcohol use.

The regression results showed that the interaction between sensation seeking and psychological control was significant, that is, the moderating effect of psychological control was significant. Finally, to further reveal the direction of the moderating effect of psychological control on the relationship between sensation seeking and tobacco and alcohol use, a simple slope test (Toothaker, 1994) was used, and an interaction map of sensation seeking and psychological control was obtained (see Figure 1).

The results of the simple slope test showed that under a low psychological control level, the predictive effect of sensation seeking on tobacco and alcohol use in junior high school students was not significant (*β* = 0.041, *t* = 0.356, *p* > 0.05). Further, the analysis of the predictive effect under a high psychological control level found that the positive predictive effect of sensation seeking on tobacco and alcohol use among junior high school students was significant (*β* = 0.155, *t* = 2.038, *p* < 0.05). This indicates that the high level of parental psychological control plays a moderating role in the relationship between junior high school students’ sensation seeking and their tobacco and alcohol use. Reducing parents’ psychological control can reduce the negative effects of sensation seeking on alcohol and tobacco use among junior high school students.

### Multiple Comparisons

**Figure 2:**
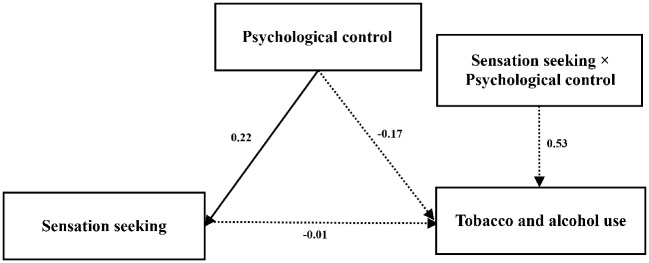
Unconstrained model of urban area.

**Figure 3:**
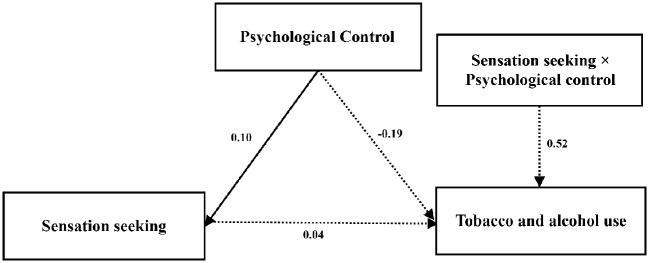
Unconstrained model of rural area.

The results of this study found that there is a significant urban-rural difference in sensation seeking and the use of tobacco and alcohol. To test the suitability of this path model graph for different regions, that is, to test its consistency for junior high school students given the demographic variables, multiple comparisons were conducted to the moderating model. The unconstrained model of urban and rural areas is shown in Figures 2,3.

The fit index of the multiple comparison models is shown in Table 5. The results showed that RMSEA < 0.1 and that CFI, GFI, and other fit indices are close to 1, indicating that the model has a good fit. *p* < 0.001 but △CFI < 0.01, it can be seen that the difference between the model M2 after limiting the regression coefficient and the model M1 without defining the difference is not significant, indicating that the urban-rural moderating effect is not significant; this is not consistent with Hypothesis 4.

**Table 5:** Multiple comparison model fitting index.

Model	*χ*^2^	df	*χ*^2^/df	GFI	AGFI	RMSEA	CFI	△CFI	*p*
Unconstrained	121.931	46	2.651	0.974	0.949	0.040	0.984	0.009	0.000
Structural weights	175.369	55	3.189	0.963	0.940	0.047	0.975		0.000

## Discussion

The present study primarily aimed to examine differences in the use of tobacco and alcohol by junior high school students under different parental control levels (including parental psychological control and parental behavioral control) and further explore the relationship between sensation seeking and alcohol and tobacco use at high and low levels of parental control. This study constructed a moderation model based on the parental effect model, social connection theory, and previous research. It is clear that sensation seeking in which conditions (regulatory role of parental control) had a predictive effect on adolescents’ tobacco and alcohol use. In addition, this study also verified the urban-rural differences in the regulation model. This study has certain theoretical and practical significance in guiding parents to adopt appropriate control methods, as well as in helping adolescents with different levels of sensation seeking to prevent and reduce tobacco and alcohol use.

### Gender, Grade, and Urban-Rural Differences in the Use of Tobacco and Alcohol in Junior High School Students

In this study, the gender difference test of junior high school students using tobacco and alcohol found that boys engage in more smoking and drinking behavior than girls, which is consistent with previous research results (Wan et al., 2009; Chen et al., 2012; Xia et al., 2012). On the one hand, because male adolescents are driven by imitative psychology, they will imitate the smoking and drinking behavior of their surrounding companions or adults. On the other hand, the ability of male adolescents to identify right and wrong is relatively low, so they cannot correctly appreciate the harmful nature of smoking and drinking. They consider these behaviors to be a symbol of maturity (Yuan et al., 2016), and some even believe that smoking and drinking are attractive to the opposite sex. The study also found that the tobacco and alcohol use in junior high school students has significant urban-rural differences, with more tobacco and alcohol use behaviors in cities than in rural areas; this is not consistent with Hypothesis 1b of the study. On the one hand, it may be because of the great difference between urban and rural parents’ education methods for teenagers. Urban teenagers’ parents often refuse and deny their children, punish them severely, and intervene excessively more than rural parents (Zhang, 1997; Li and Wang, 2015), making teenagers use alcohol and tobacco to vent their inner dissatisfaction. On the other hand, urban junior high school students have more contact with new stimuli and temptations and may have more opportunities to obtain tobacco and alcohol. Therefore, urban students show more tobacco and alcohol use behaviors. There is no significant grade difference in the use of tobacco and alcohol in junior high school students, which is consistent with the research results of Wang et al. (2013) and the research hypothesis of this study, that is, the difference in the use of tobacco and alcohol among junior high school students in the first and second grades is not significant. One possible explanation is that grade 7 and grade 8 students are of similar age, have a similar level of psychological development, and a similar ability to distinguish right from wrong. Meanwhile, they also have roughly a similar level of understanding about the harm caused by tobacco and alcohol use. Therefore, there is no significant grade difference in the use of tobacco and alcohol.

### The Predictive Effect of Sensation Seeking on Alcohol and Tobacco Use

The results of this study indicated that there is a significant positive correlation between sensation seeking and the use of tobacco and alcohol among junior high school students. Sensation seeking can predict the use of tobacco and alcohol in junior high school students, which is consistent with previous research results (Ye et al., 2011; Yuan et al., 2016). Impulsivity is a risk factor for tobacco and alcohol use, and as one of the impulsivity personality traits, sensation seeking has a significant positive correlation with tobacco and alcohol use (Gonzalez et al., 2011). As previously mentioned, tobacco and alcohol use together can make individuals happier, thus teenagers with a high level of sensation seeking will take the initiative to smoke and drink to meet their need for stimulation. The results of this study suggest that adolescents with high sensation-seeking levels may be more likely to use alcohol and tobacco for stimulus-seeking or rule-breaking reasons than adolescents with low sensation-seeking levels. This study proved that sensation seeking was a risk factor for junior high school students’ tobacco and alcohol use and verified the important influence of sensation seeking on their substance use. Parents and schools should pay more attention to the behavior of junior high school students with high sensation-seeking levels, guide them to choose appropriate activities to meet their need for thrill-seeking, and help them to avoid or reduce externalized behaviors such as smoking and drinking.

### The Moderating Role of Parental Psychological Control

In this study, parental control was investigated as a moderating variable between sensation seeking and the use of tobacco and alcohol. The results showed that parental psychological control plays a moderating role in and can enhance the relationship between junior high school students’ sensation seeking and their use of tobacco and alcohol, which verifies the hypothesis of this study and conforms to the parental effect model. This research result is similar to relevant research conclusions within China and worldwide; namely, a high level of psychological control is related to externalized problem behaviors among children and adolescents (Arim and Shapka, 2008; Gao et al., 2016; Yu, 2016; Xing et al., 2017), which may lead them to engage in smoking, drinking, drug use, Internet addiction, aggressive behaviors, and other problems (Ye et al., 2012; Cui et al., 2014; Lai et al., 2014a). This result is not difficult to understand. Parental psychological control destroys children’s psychological autonomy by stimulating guilt and withdrawing care (Barber, 1996), which is a negative control method. Eventually, teenagers’ basic psychological needs (such as autonomy needs and relationship needs) cannot be satisfied. Previous studies have shown that teenagers meet their basic psychological needs through alcohol and tobacco use (Niemiec et al., 2009; Gillison et al., 2011; Xia and Ye, 2014). Therefore, high levels of parental psychological control can enhance the relationship between sensation seeking and the alcohol and tobacco use.

Previous studies have found that low levels of parental behavioral control can significantly predict a higher level of externalization behaviors among adolescents, such as antisocial behavior, drug abuse, and disciplinary behavior (Li et al., 2003; Pettit et al., 2010; Xiao, 2016). However, in this study, it was found that parental behavioral control was not significantly related to tobacco and alcohol use, which is similar to the results of previous studies; that is, the correlation between behavioral control and junior high school students’ externalization is not significant (Wang et al., 2013). First, this may be caused by the different views of teenagers on parental behavior control between Chinese and western cultural backgrounds. In the context of Chinese collectivistic culture, children tend to regard their parents’ harsh discipline as an expression of caring and loving (Wang and Liu, 2018). Parental behavioral control, such as corporal punishment, may be commonly accepted by children (Wang and Liu, 2014); regardless of the degree of parental control, we think that parental behavioral control may have less effect on children’s externalizing behavior and have no significant differences in China. Second, the reason for this lack of correlation may be that teenagers from different educational backgrounds have different views on parental behavioral control. Parental rearing patterns have a wide-ranging impact on the psychological development and psychological barriers of children and adolescents (Hiramura et al., 2010), affecting the personality of children and adolescents (Jiang et al., 2013). Adolescents who live under an authoritative and strict family atmosphere are more likely to have problems with conduct, hostile psychological barriers, and negative and hostile attitudes toward parental behavioral control. Therefore, the higher the level of parental behavioral control is, the stronger the child’s anger and resistance, and the likelier that they will engage in problem behaviors such as alcohol and tobacco use that are not accepted by parents. Teenagers who live in a democratic and harmonious family atmosphere are more likely to regard parental behavioral control as a manifestation of their parents’ care and to feel gratitude for their parents’ efforts, thus generating more adaptive behaviors (Zou et al., 2010).

### Multiple Comparisons

To investigate the moderating effect of urban-rural demographic variables on this model of parental psychological control, this study conducted a multi-group comparative analysis. The results showed that the moderating effect of urban and rural areas was not significant; that is, for adolescents residing in both urban and rural areas, the moderating effect of parental psychological control on the influence of sensation seeking over the use of tobacco and alcohol in junior high school students was basically stable. The reasons are as follows. First, urban and rural parents have roughly the same psychological control over junior middle school students, for example, engaging in guilt or love cancellation (Barber, 1996). This result suggests that neither urban nor rural parents should engage in higher levels of psychological control of junior high school students, such as not giving them space for independent thinking or for determining the correct choices, or apply methods to psychologically control their children. Instead of helping their children’s growth, ultimately, it will only backfire and lead to more problem behaviors such as alcohol and tobacco use (Chen et al., 2018). Second, with the popularization and development of the Internet, rural and urban children are exposed to roughly the same information, and thus they form similar outlooks on life and values. Third, the withdrawal of rural schools and the process of urban-rural integration have narrowed the gap between rural and urban areas. Children born in rural areas have the opportunity to study in urban or urban-rural areas. Finally, the state is paying increasing attention to education, and thus it provides excellent teachers for rural schools, encourages urban teachers to go to the countryside, and promotes the development of rural cultural education.

## Contributions and Limitations

There are some shortcomings in the current study. First, the cross-sectional design used in this study may have a generation effect, which to some extent weakens the credibility of the causal relationship between the inferred variables. Second, this study adopts the “self-report method” with teenagers and a questionnaire for measurement. This research method is relatively simple. In the future, this research can be further verified by combining an experimental task with the questionnaire. In addition, the students’ emphasis on parental communication and understanding deviation may affects the direction and effect of parental control, are also worth considering factors in the use of tobacco and alcohol. Teenagers with high sensitivity will pay more attention to their parents’ words, may be more likely to report more internalizing and externalizing problems if they perceived parental control as a signal that they are being rejected by their parents. So in the future research, we should focus on more additional mediating and moderating factors between sensory seeking and the use of tobacco and alcohol. Finally, this study focuses on the use of tobacco and alcohol among junior high school students in grades 7 and 8. For high school students and college students, this study did not examine or verify either the role of psychological control or the regulatory role of behavioral control in the influence of sensation seeking on the use of tobacco and alcohol among other groups. To further enrich research on the use of tobacco and alcohol and create a healthy and harmonious living environment for students, future research should explore these problems.

Despite these limitations, the present study provides some valuable information to the related literature and has important practical implications. Based on the ecological systems theory, this study introduced the family variable of parental control (including parental psychological control and parental behavioral control), discussed the moderating effect on sensory seeking and the use of alcohol in junior middle school students. The results show that parental psychological control plays a moderating role in the relationship between sensory seeking and the use of tobacco and alcohol, providing ideas for avoiding and reducing the use of tobacco and alcohol in junior high school students. First, attention should be paid to the influence of sensation seeking on alcohol and tobacco use among adolescents. On the one hand, in adolescence, individuals’ sensation-seeking level increases rapidly with age (Steinberg et al., 2008). Therefore, it is necessary to understand teenagers’ sensation-seeking level over time, guide them to meet their needs for stimulating experiences through appropriate activities and channels, and enable them to find release and satisfaction through reasonable and appropriate channels. On the other hand, attention should be paid to intervention around sensation seeking. Sensation seeking is relatively plastic (Bardo et al., 1996), and intervention in adolescents’ sensation seeking can help reduce problems such as alcohol use. Second, this study found that parental psychological control will enhance the influence of sensation seeking on tobacco and alcohol use, so we should also aim at improving communication between teenagers and their parents to prevent or reduce excessive psychological control and improve the psychological quality of their environmental response. Psychological quality helps teenagers to live actively. Third, this model is applicable to both urban and rural areas in China, which enlighten us not to overstate the differences between urban and rural areas in view of some problems. Whether in urban or rural areas, reducing parental psychological control is an effective way to reduce the use of tobacco and alcohol in adolescents.

## Data Availability

The raw data supporting the conclusions of this manuscript will be made available by the authors, without undue reservation, to any qualified researcher.

## Ethics Statement

This study was carried out in accordance with the recommendations of the Institutional Review Board of Shandong Normal University with written informed consent from all subjects in accordance with the Declaration of Helsinki. The protocol was approved by the Institutional Review Board of Shandong Normal University.

## Author Contributions

FX and WD collected and analyzed the data under the supervision of WZ. WZ designed the study and contributed to materials and analysis tools. WZ, FX, WD, YS, and QZ contributed to the writing of the manuscript. FX, WD, YS, and QZ contributed to the revision. YS, FX, and WD revised the manuscript and replied to comments.

### Conflict of Interest Statement

The authors declare that the research was conducted in the absence of any commercial or financial relationships that could be construed as a potential conflict of interest.
